# Supplementary material for: Structure of SARS-CoV-2 membrane protein essential for virus assembly
Source: Nat Commun. 2022 Aug 5;13:4399. doi: 10.1038/s41467-022-32019-3 (PMC9355944; doi:10.1038/s41467-022-32019-3)
Supplement: Supplementary file 1 — Supplementary Information [file 41467_2022_32019_MOESM1_ESM.pdf]

Supplementary Information

Structure of SARS-CoV-2 membrane protein essential for virus assembly

Authors

Zhikuan Zhang[1], Norimichi Nomura[2], Yukiko Muramoto[3,4,5], Toru Ekimoto[6], Tomoko Uemura[2], Kehong Liu[2], Moeko Yui[1], Nozomu Kono[1], Junken Aoki[1], Mitsunori Ikeguchi[6,7], Takeshi Noda[3,4,5], So Iwata[2,8], Umeharu Ohto[*1] and Toshiyuki Shimizu[*1]

Affiliations

1. Graduate School of Pharmaceutical Sciences, The University of Tokyo, 7-3-1 Hongo, Bunkyo-ku, Tokyo 113-0033, Japan
2. Department of Cell Biology, Graduate School of Medicine, Kyoto University, Yoshida Konoe-cho, Sakyo-ku, Kyoto 606-8501, Japan.
3. Laboratory of Ultrastructural Virology, Institute for Frontier Life and Medical Sciences, Kyoto University, 53 Shogoin Kawahara-cho, Sakyo-ku, Kyoto 606-8507, Japan.
4. Laboratory of Ultrastructural Virology, Graduate School of Biostudies, Kyoto University, 53 Shogoin Kawahara-cho, Sakyo-ku, Kyoto 606-8507, Japan.
5. CREST, Japan Science and Technology Agency, 4-1-8 Honcho, Kawaguchi, Saitama 332-0012, Japan.
6. Computational Life Science Laboratory, Graduate School of Medical Life Science, Yokohama City University, 1-7-29, Suehiro-cho, Tsurumi-ku, Yokohama, Kanagawa 230-0045, Japan
7. HPC- and AI-driven Drug Development Platform Division, Center for Computational Science, RIKEN, Yokohama 230-0045, Japan
8. RIKEN SPring-8 Center, Kouto, Sayo-cho, Sayo-gun, Hyogo 679-5148, Japan.

*Corresponding author:
Email: shimizu@mol.f.u-tokyo.ac.jp (T.S.); umeji@mol.f.u-tokyo.ac.jp (U.O.)

This file contains Supplementary Figures 1-14 and Supplementary Table1:

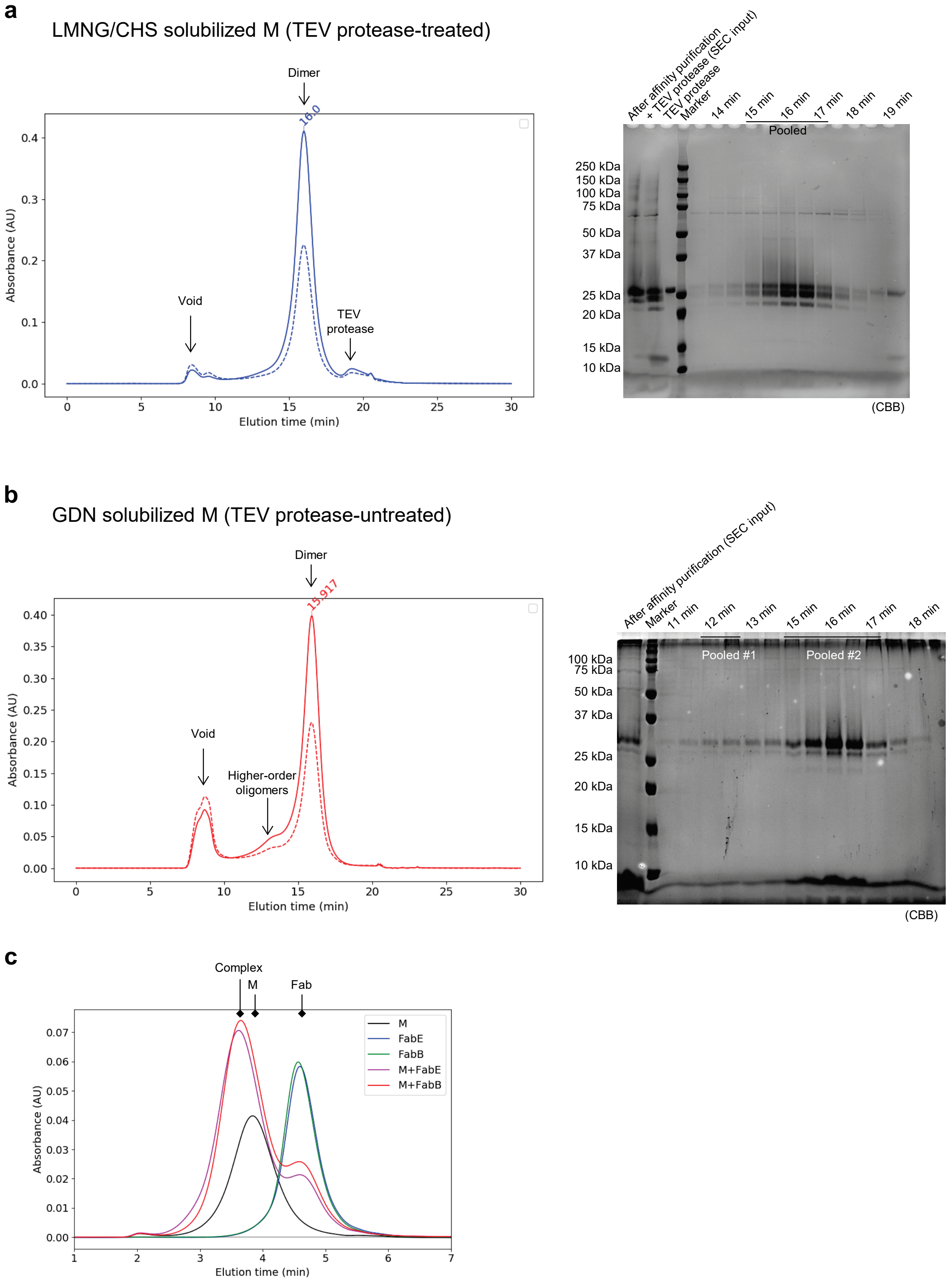

Supplementary Fig. 1 Sample preparation of SARS-CoV-2 M protein

a, **b,** Representative size-exclusion chromatography (SEC) profiles (left) of LMNG/CHS-solubilized M protein (**a**) and GDN-solubilized M protein (**b**) and SDS-PAGE analysis of the SEC fractions stained with Coomassie blue (CBB) (right). M protein contains a single *N*-glycosylation site in its N-terminal region (N5), and SDS-PAGE analysis showed two separated bands, indicating heterogeneity in *N*-glycosylation. The purification was repeated three times for LMNG/CHS-solubilized protein or twice for GDN-solubilized protein with similar results. Source data are provided as a Source Data file. **c**, SEC analysis of M protein and Fab (YN7756_1, Fab-E; YN7717_9, Fab-B) binding. Absorbances at 280 nm and 260 nm are shown as solid and dashed lines, respectively.

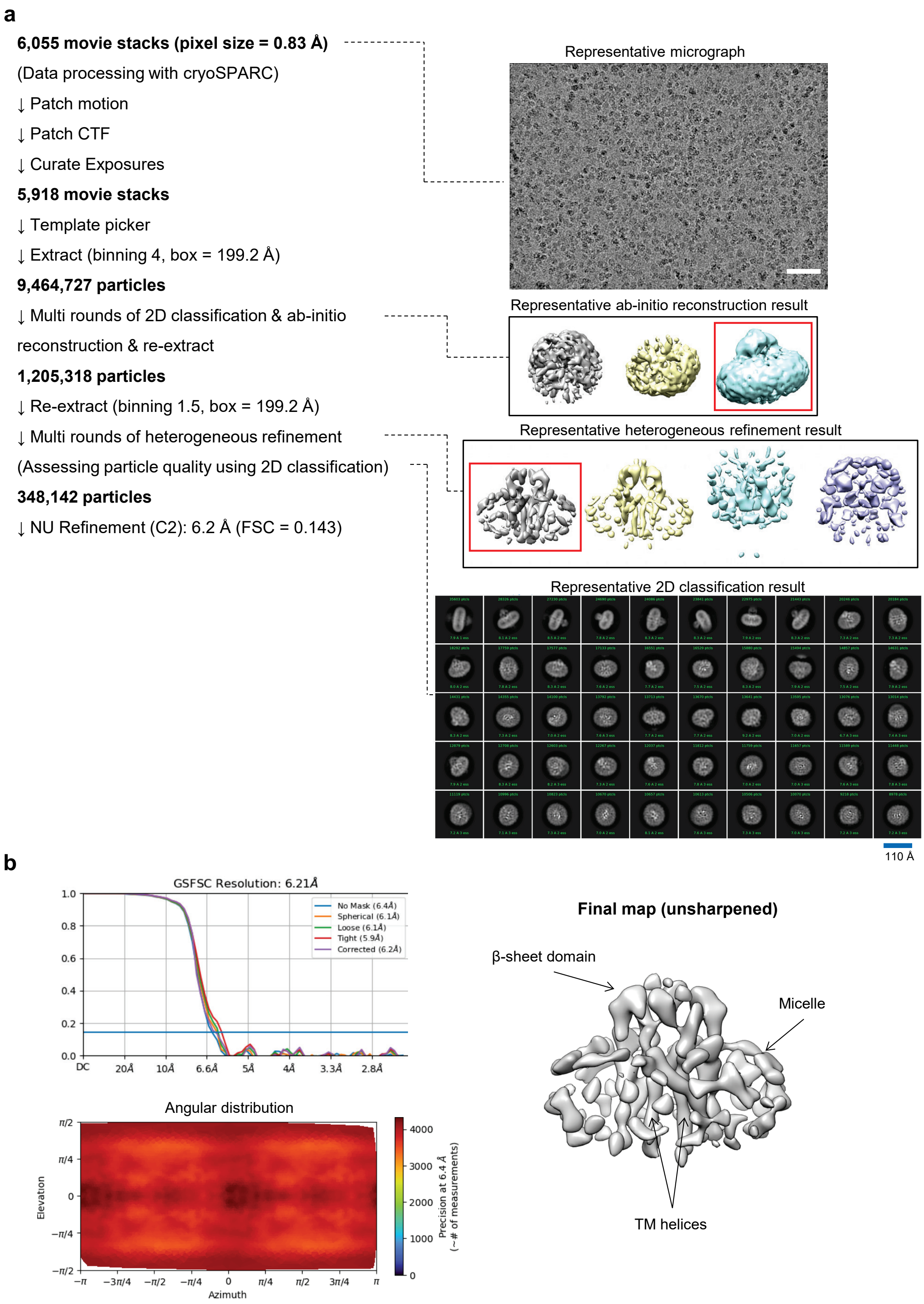

Supplementary Fig. 2 Cryo-EM analysis of the LMNG/CHS-solubilized M protein dimer

a, Image processing workflow of cryo-EM analysis of the LMNG/CHS-solubilized M protein dimer without antibody. Representative motion-corrected micrograph (white bar, 50 nm), 2D class average images, ab initio reconstruction results, and heterogeneous refinement results are shown. The good classes selected for further processing are indicated by red squares. **b**, Gold-standard FSC curve of the final 3D reconstruction (resolution cut-off at FSC = 0.143), angular distribution of refined particles, and the final unsharpened 3D map are shown. Although 2D class average images showed clear secondary structural features of the M protein dimer, the 3D reconstruction yielded only a low-resolution 3D map with the slightly recognisable densities of the TM helices and the strong densities of the micelle region.

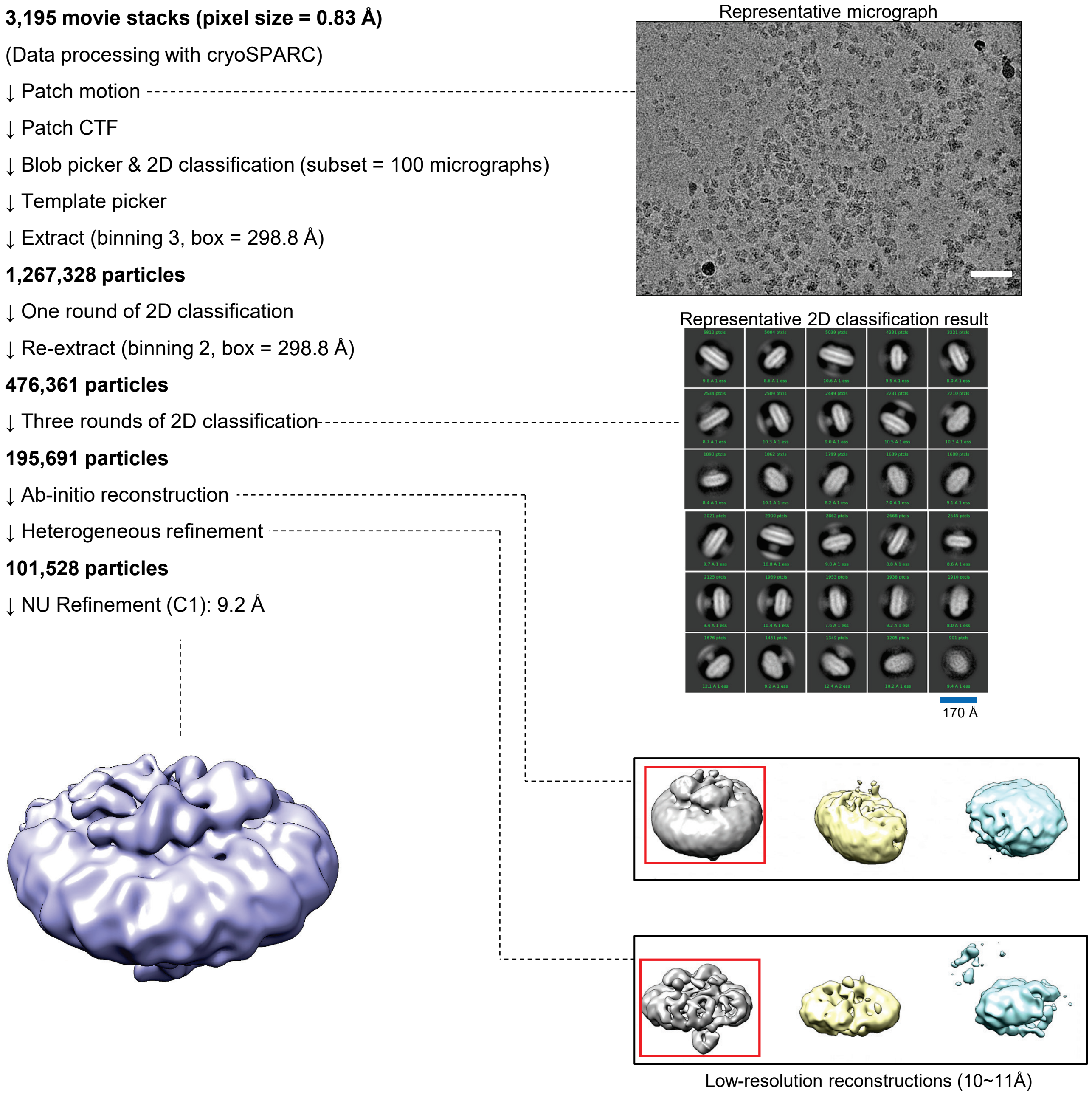

Supplementary Fig. 3 Cryo-EM analysis of the GDN solubilized M protein oligomer

Image processing workflow of cryo-EM analysis of the GDN solubilized M protein oligomer. Representative motion-corrected micrograph (white bar, 50 nm), 2D class average images, ab-initio reconstruction result, heterogeneous refinement result, and low-resolution final 3D map are shown. The classes selected for further processing are indicated by red squares. Although this data gave only a low-resolution reconstruction, higher-order oligomers of the M protein could be clearly observed in 2D class average images.

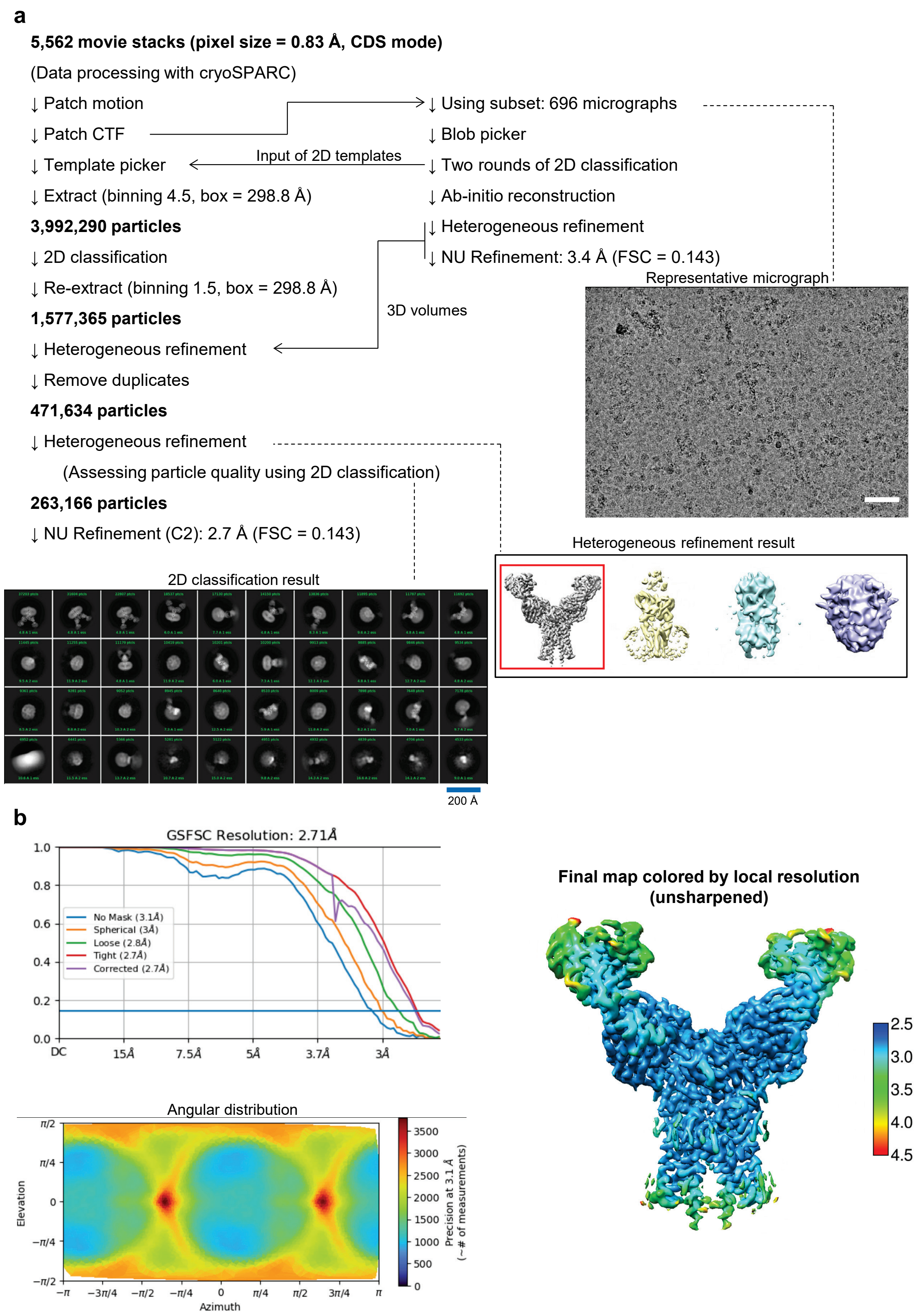

Supplementary Fig. 4 Cryo-EM analysis of M/Fab-E complex

a, Image processing workflow of cryo-EM analysis of M/Fab-E complex. Representative motion-corrected micrograph (white bar, 50 nm), 2D class average images, and heterogeneous refinement results are shown. Good classes selected for further processing are indicated by a red square. **b**, Gold-standard FSC curve of the final 3D reconstruction (resolution cut-off at FSC = 0.143), angular distribution of refined particles, and the final unsharpened 3D map coloured according to the local resolution are shown.

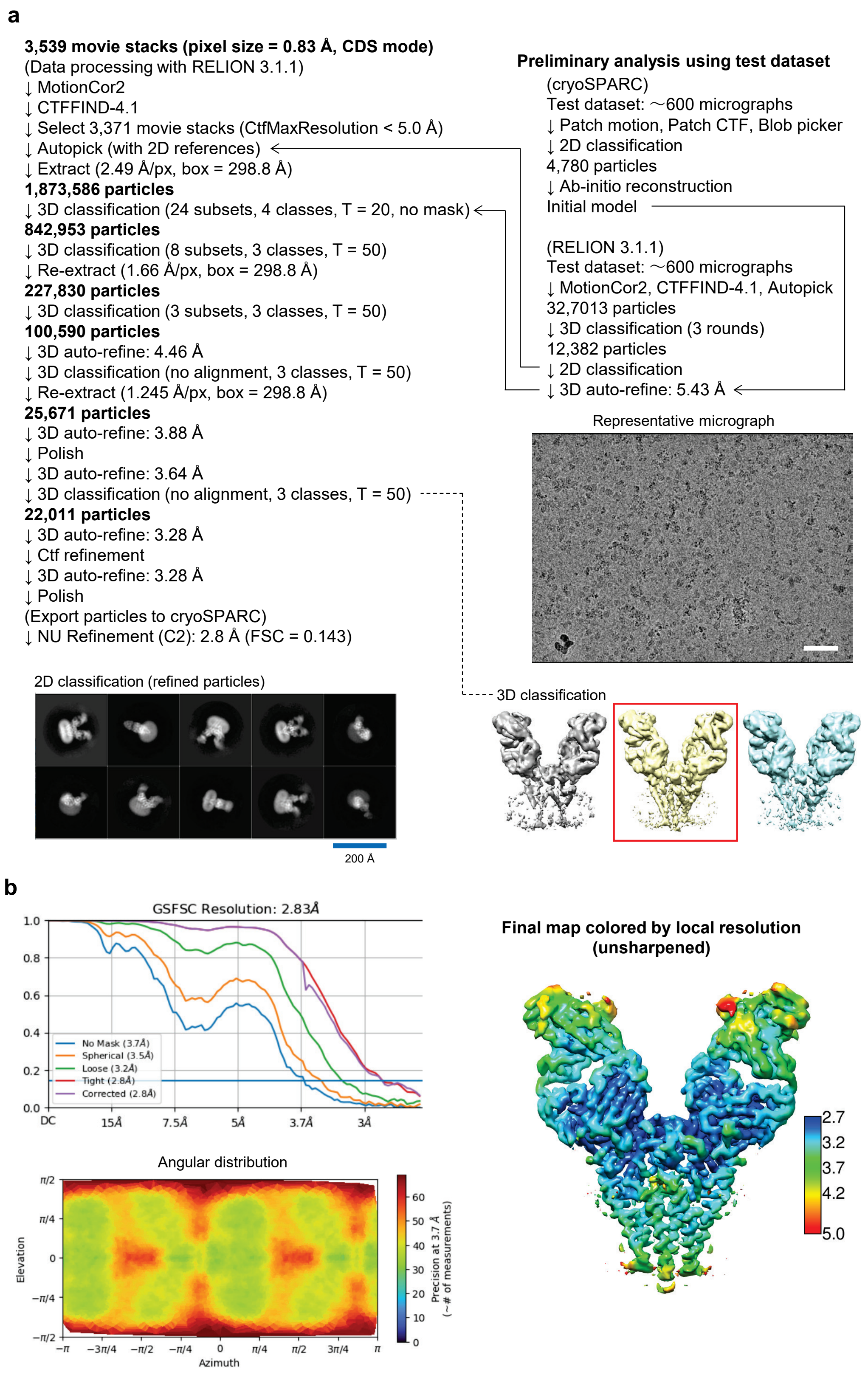

Supplementary Fig. 5 Cryo-EM analysis of M/Fab-B complex

a, Image processing workflow of cryo-EM analysis of M/Fab-B complex. Representative motion-corrected micrograph (white bar, 50 nm), 2D class average images, and 3D classification results are shown. The good class selected for further processing is indicated by a red square. **b**, Gold-standard FSC curve of the final 3D reconstruction (resolution cut-off at FSC = 0.143), angular distribution of refined particles, and the final unsharpened 3D map coloured according to the local resolution are shown.

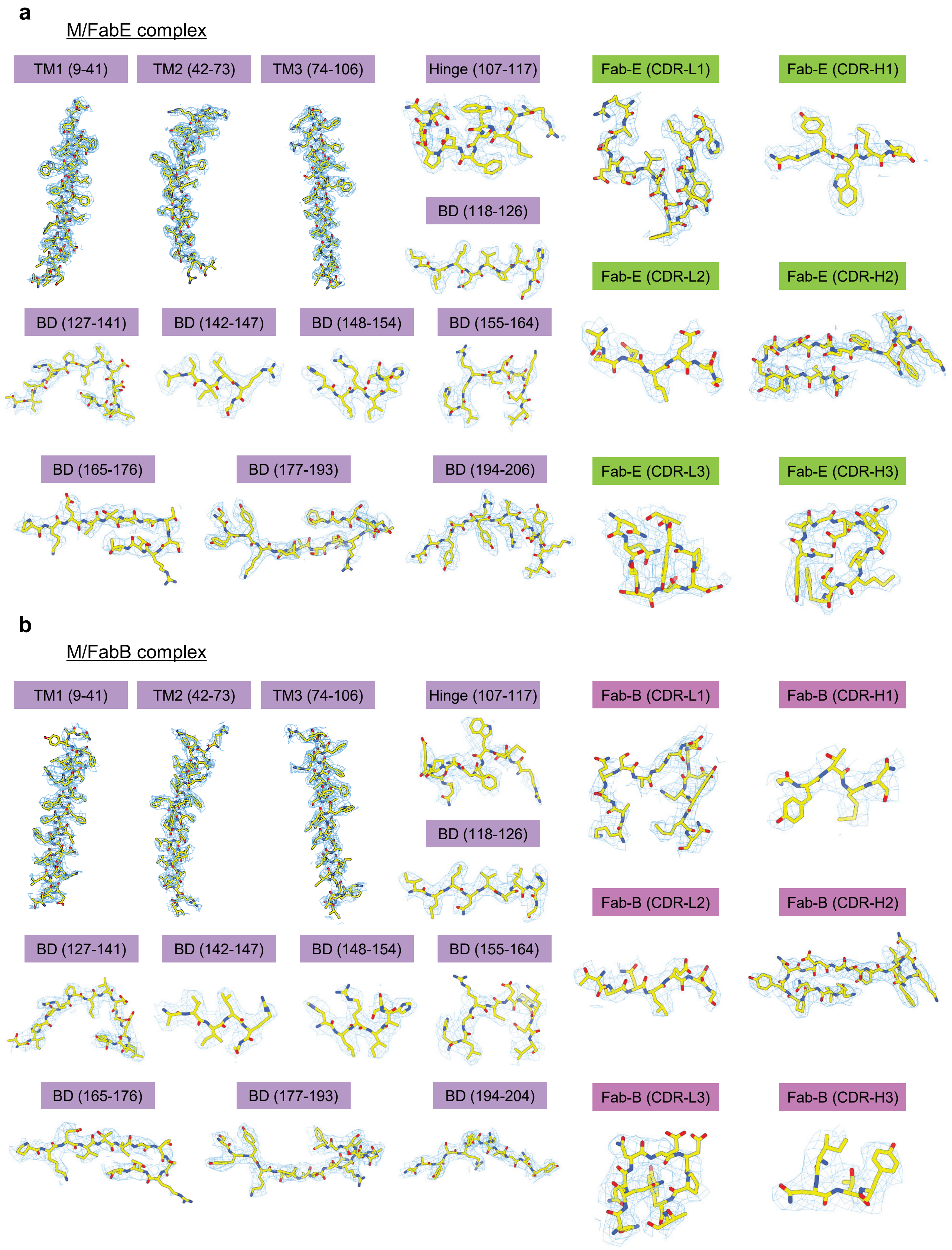

Supplementary Fig. 6 Comparison between atomic models and cryo-EM density maps
Cryo-EM density maps of the M/Fab-E complex (**a**) and the M/Fab-B complex (**b**) around each TM helix, the hinge region, the structural motifs in the BD of the M protein, and the complementarity determining regions (CDRs) of Fab are shown.

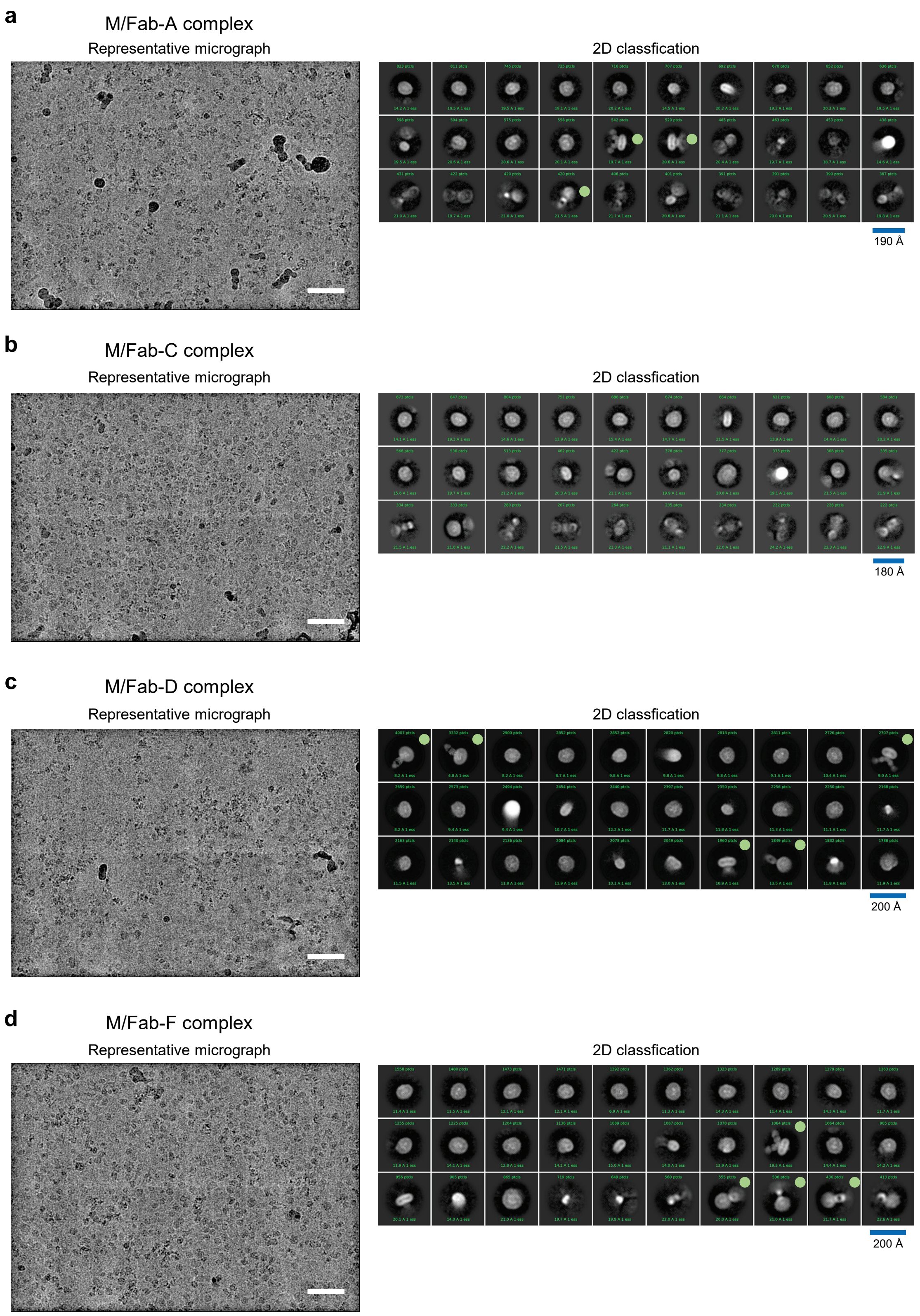

Supplementary Fig. 7 Cryo-EM analysis of four M/Fab complexes

Representative motion-corrected cryo-EM micrographs (white bar, 50 nm) (left) and 2D class average images (right) of the M/Fab-A (66 micrographs) (**a**), M/Fab-C (59 micrographs) (**b**), M/Fab-D (551 micrographs) (**c**), and M/Fab-F (102 micrographs) (**d**) complexes. Each dataset was collected from a single grid. 2D class average images with clear binding of Fab are indicated by green dots. All M/Fab complexes, except M/Fab-C, showed clear binding to the BD of the M protein dimer.

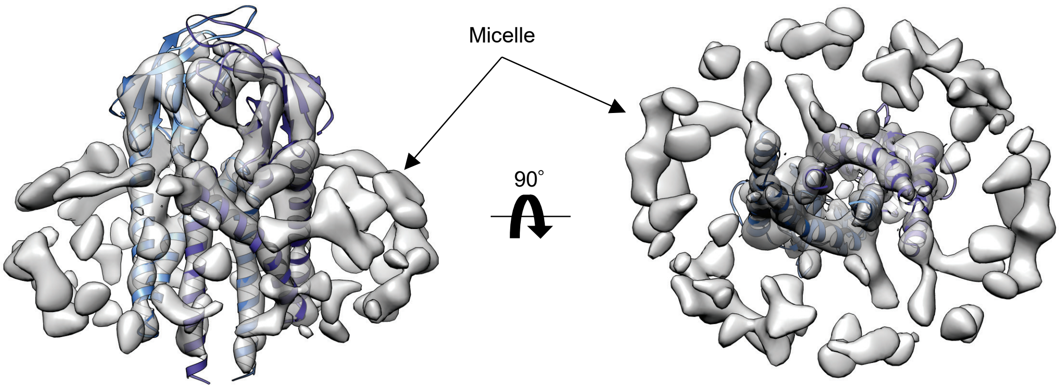

Supplementary Fig. 8 Conformation of the Fab-unbound M-protein dimer
Comparison of the long form of M protein dimer with the low-resolution 3D map of the LMNG/CHS-solubilized M protein dimer. The structure was fitted to a 3D map using Chimera.

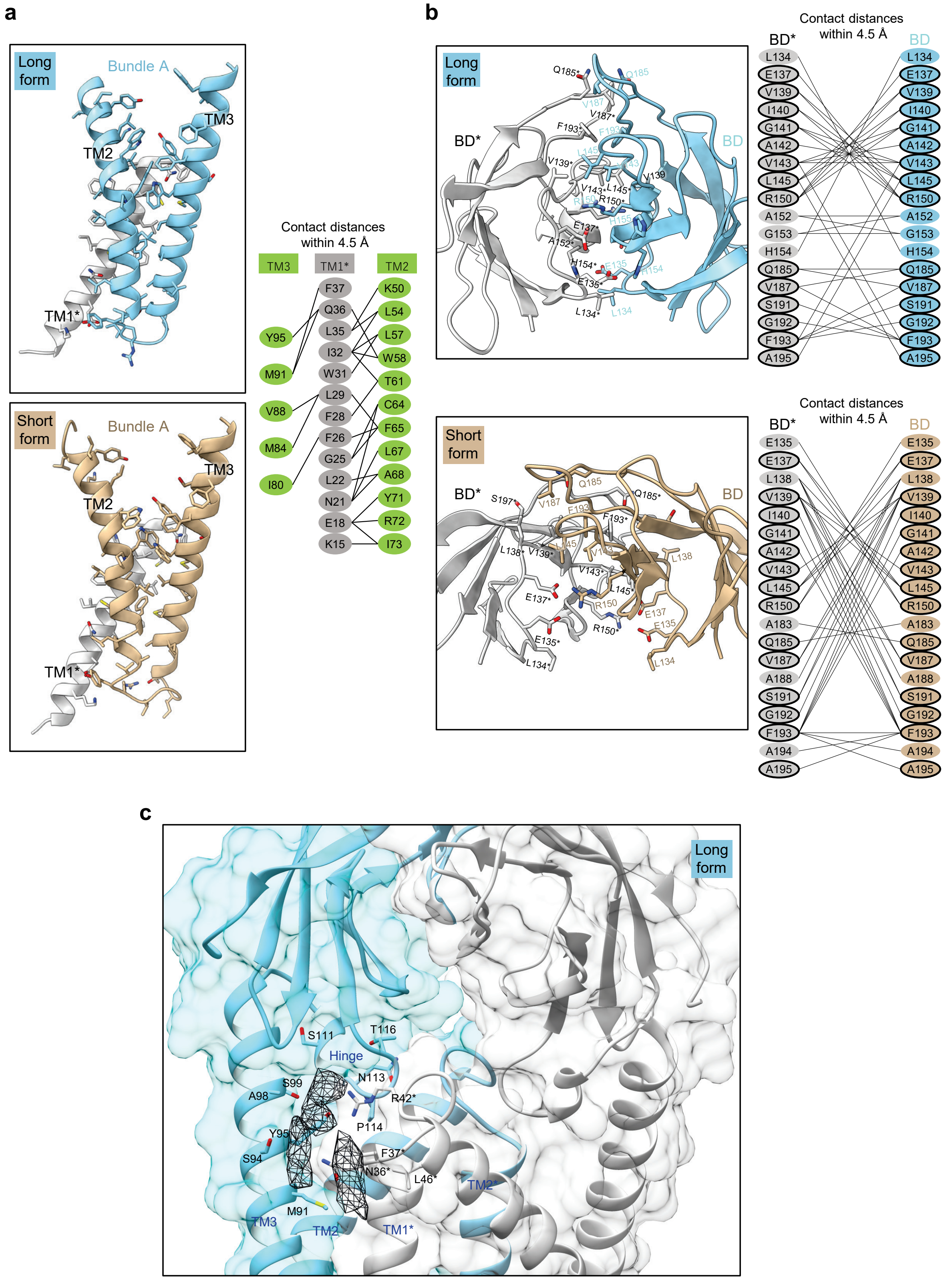

Supplementary Fig. 9 Views of the bundle, BD-BD interface and densities of phospholipid

a, **b**, Close-up view of the three-helix bundle (**a**) and the BD–BD dimerization interface (**b**) of the long form (upper) and the short form (lower). Interaction maps (contact within 4.5 Å) of the three-helix bundle and the two types of BD–BD dimerization interface are shown. **c**, Densities of ordered phospholipid molecules in the M/Fab-E complex (long form) are shown in grey mesh (level = 0.30). Residues near the densities are shown using stick representations. The M protein dimer is shown in the cartoon as a transparent surface representation.

a

Species	Uniprot Entry ID	Sequence identity to SARS-CoV-2 M protein
SARS-CoV-2	P0DTC5	-
Bat coronavirus RaTG13	A0A6B9WDP7	99.55%
Pangolin coronavirus	A0A6G6A1P9	98.20%
SARS-CoV	P59596	90.54%
Bat coronavirus HKU3	Q3LZX9	89.95%
Bat Hp-betacoronavirus	A0A088DIE6	52.94%
MERS-CoV	A0A678TA40	45.05%
Hedgehog coronavirus 1	A0A4D6G4C3	44.55%
Murine coronavirus	P03415	36.68%

b

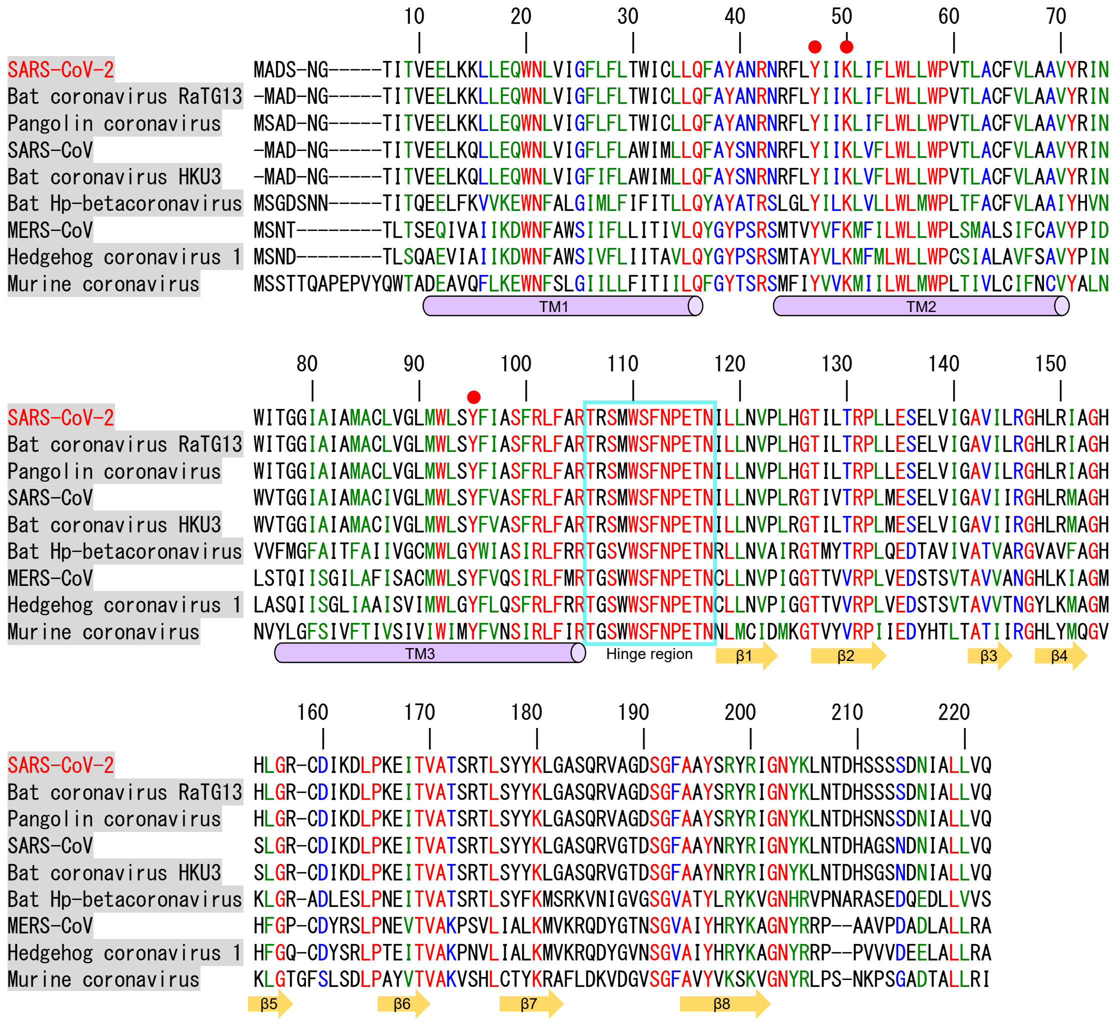

Supplementary Fig. 10 Sequence alignment of *Betacoronavirus* M proteins

Sequence identities (**a**) between SARS-CoV-2 M protein and related *Betacoronavirus* M proteins and sequence alignment (**b**) of M proteins from nine *Betacoronaviruses.* The alignment was performed using the PRABI multiple sequence alignment CLUSTALW web server. Residues are coloured to indicate the degree of similarity: red residues display the highest similarity, followed by green, blue, and then black (lowest similarity). TM helices and β-sheets are indicated by purple and orange arrows, respectively. The highly conserved hinge region is boxed with cyan lines. Conserved residues (Y47, K50, and Y95 in the SARS-CoV-2 M protein) are indicated by blue triangles.

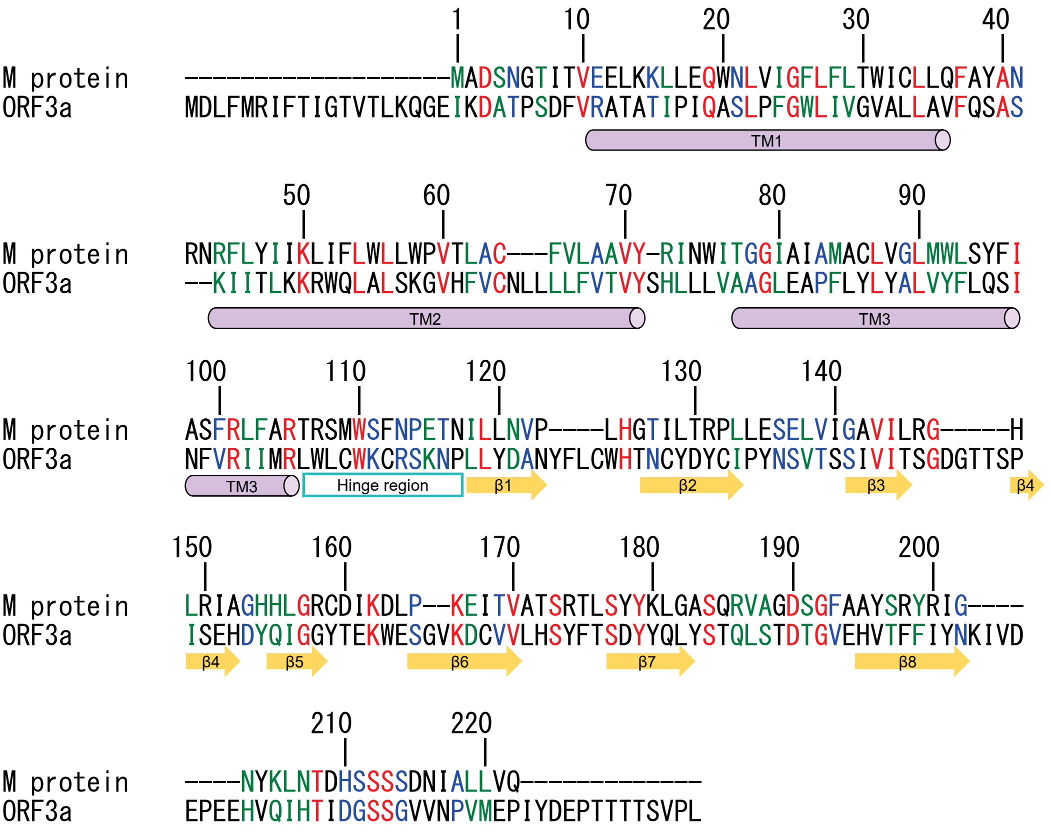

Supplementary Fig. 11 Sequence alignment of SARS-CoV-2 M protein and ORF3a
Sequence alignment of SARS-CoV-2 M protein (UniProt: P0DTC5) and ORF3a (UniProt: P0DTC3). The alignment was performed using the PRABI multiple sequence alignment CLUSTALW web server. Residues are coloured to indicate the degree of similarity: red residues display the highest similarity, followed by green, blue, and then black (lowest similarity).

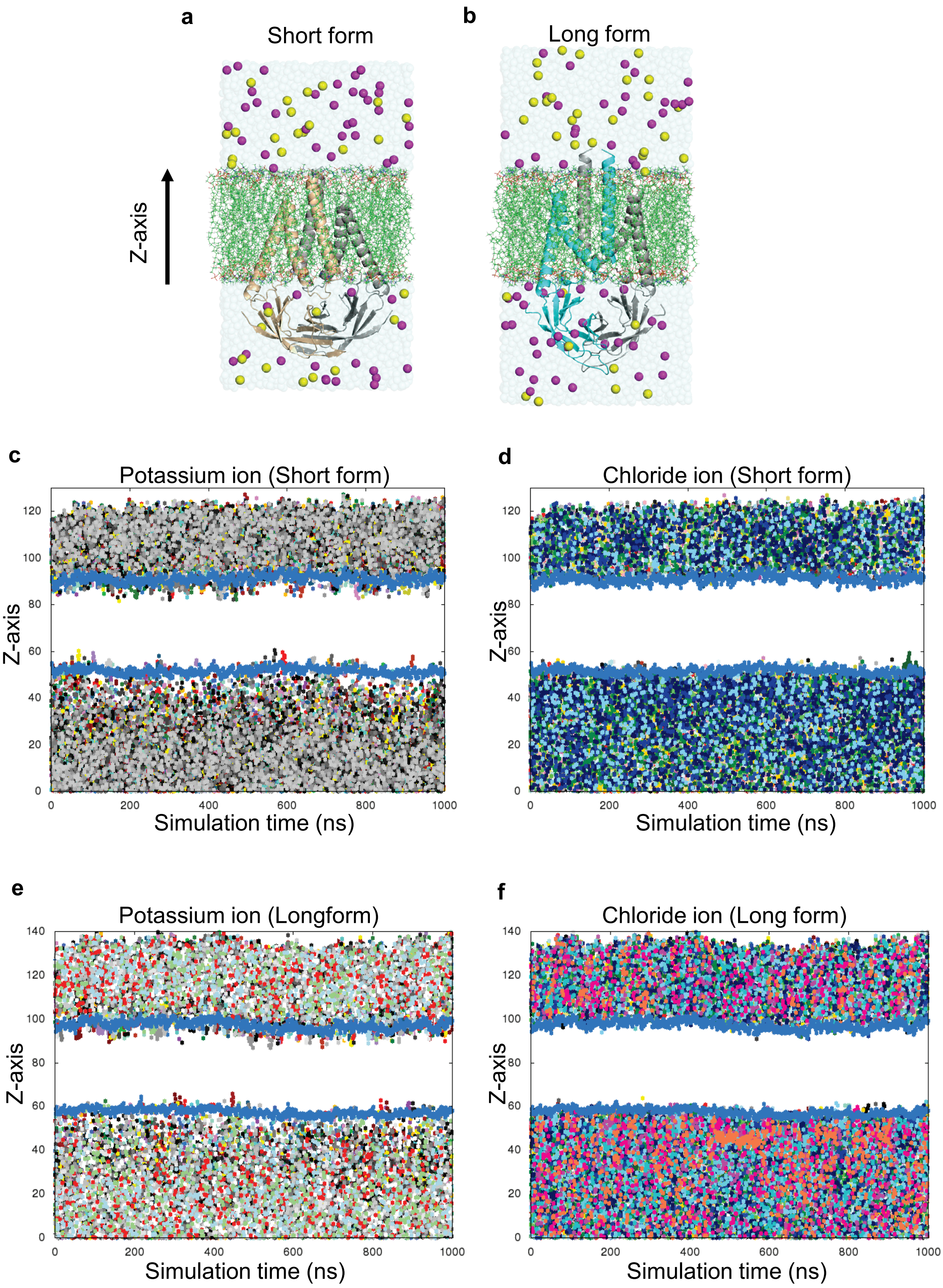

Supplementary Fig. 12 MD simulation

a, b, Initial membrane–water systems for the short form (**a**) and long form (**b**) of the M protein. The M protein dimers are shown as cartoon representations, the POPC membrane is shown using line representations, and the water molecules, potassium ions, and chloride ions are shown as cyan-, yellow-, and magenta-colored spheres, respectively. The z-axis is defined so that the axis is perpendicular to the membrane surface. **c–f,** Trajectories of ions along the z-axis during MD simulations with an external electric field. The averaged positions of the phosphorus atoms in the membrane molecules of the upper and lower leaflets are plotted as cyan points. Other plots are the positions of the potassium and chloride ions during MD simulations of the short form (**c**, **d**) and long form (**e**, **f**).

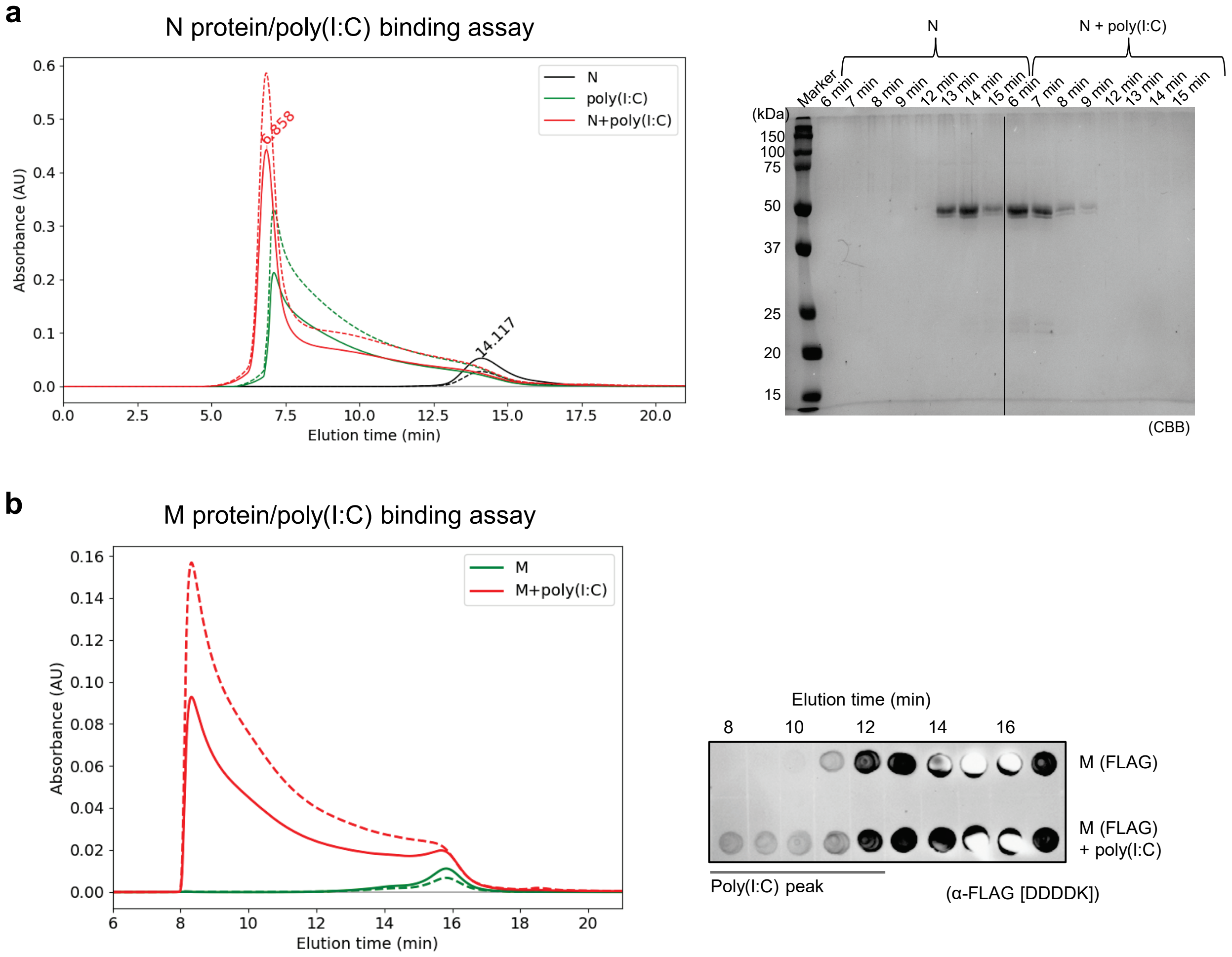

Supplementary Fig. 13 SEC analysis of poly(I:C) binding

a, Binding assay of N protein and poly(I:C) in SEC. Absorbances at 280 nm and 260 nm are shown as solid and dashed lines, respectively. N protein alone, poly(I:C) alone, and N protein + poly(I:C) are shown as black, green, and red lines, respectively. Eluted fractions of N protein alone and N protein + poly(I:C) were analysed by SDS-PAGE (stained with CBB). The experiment was repeated twice with similar results. Source data are provided as a Source Data file. **b**, Binding assay of M protein and poly(I:C) in SEC. Absorbances at 280 nm and 260 nm are shown as solid and dashed lines, respectively. M protein alone and M protein + poly(I:C) are shown as green and red lines, respectively. Eluted fractions were analysed by dot blot detection using an anti-FLAG antibody. The experiment was repeated twice with similar results. Source data are provided as a Source Data file.

a

Fab-E (Long form)

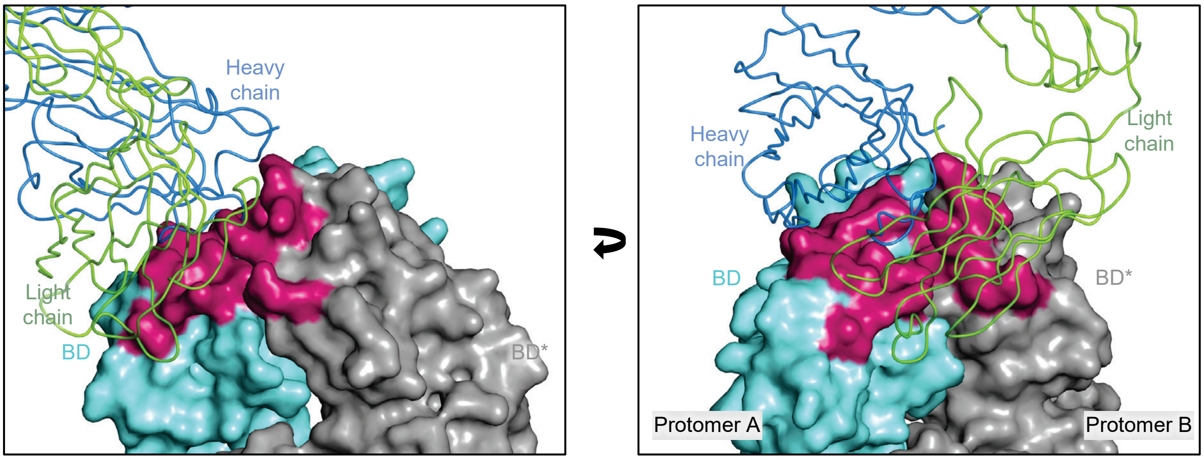

b

Fab-B (Short form)

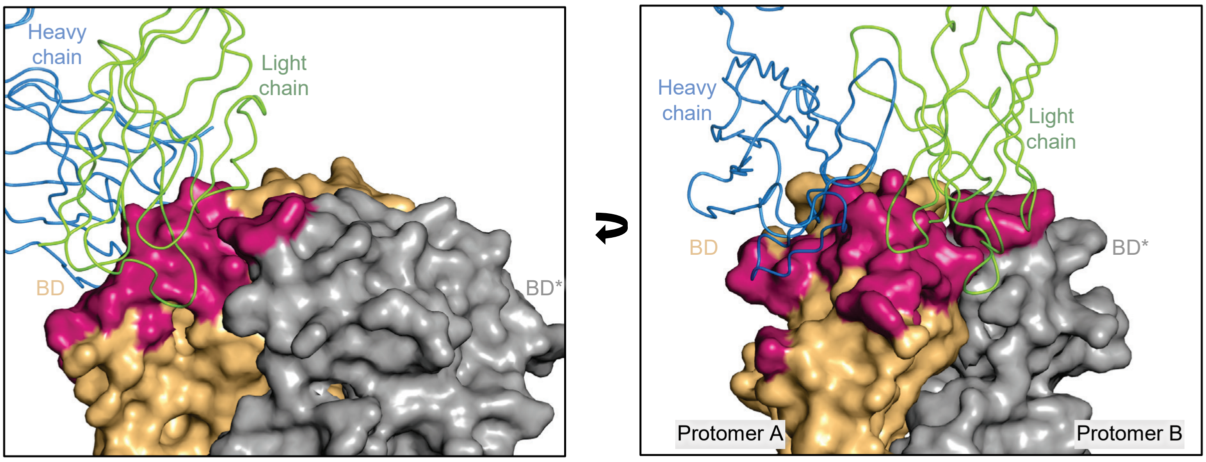

c

	Long form	Short form
Fab binding sites in protomer A	L138, V139, P165, K180, L181, G182, A183, S184, F193, S197, R198, Y199, I201	R107, P123, G126, T127, I128, L138, P165, K166, I168, T175, K180, S197, R198, Y199, R200, I201, G202, N203, Y204
Fab binding sites in protomer B	L145, R146, H148, R158, A188, G189, D190, S191	Q185, R186, V187, A188, G189

Supplementary Fig. 14 Antibody binding sites

a, **b**, The interfaces between M protein (long form) and Fab-E (**a**) and between M protein (short form) and Fab-B (**b**). The M protein and Fab are shown as surface representations and wire models, respectively. The interface residues of M protein are colored warmpink. **c**, List of detailed residues of the Fab-E and Fab-B bindings sites in the long form and the short form, respectively. Shared residues in both binding sites are underlined.

Supplementary Table 1. Statistics for data collection and structural refinement

**Data**	M protein (LMNG/CHS)	M protein (LMNG/CHS) + Fab-E	M protein (LMNG/CHS) + Fab-B
**EMDB ID**	EMD-31976	EMD-31977	EMD-31978
**PDB ID**	Not applicable	7VGR	7VGS
**Data collection and processing**			
Microscope / Voltage (kV)	Titan Krios / 300		
Detector	K3	K3 (CDS mode)	K3 (CDS mode)
Magnification	105,000		
Pixel size (Å)	0.83		
Total dose ($e^-/Å^2$) / Frames per movie	57.4 / 60	61.9 / 64	61.4 / 64
Total movie stacks	6,055	5,562	3,519
Final particle images (no.)	348,142	263,166	22,011
Symmetry imposed	C2	C2	C2
Map resolution (Å)	6.2	2.7	2.8
FSC threshold	0.143	0.143	0.143
Map sharpening B factor ($Å^2$)	Not applicable	-50.0	-40.0
**Refinement**			
Software	Not applicable	COOT, Chimera, Phenix	
Model resolution (Å) (FSC threshold=0.5/0.143)		3.0/2.6	3.4/2.8
Model composition			
Protein chains		6	6
Residues		1,272	1,254
Average B-factors ($Å^2$)		77.3	100.6
R.m.s deviations		-	
Bond lengths (Å)		0.002	0.002
Bond angles (°)		0.57	0.67
Validation			
Molprobity score		1.81	1.96
Clashscore		7.72	9.73
Poor rotamers (%)		0.18	0.38
Ramachandran plot			
Favored (%)		94.2	93.0
Allowed (%)		5.6	7.0
Outliers (%)		0.2	0.0
