# Supplementary material for: Structure of SARS-CoV-2 membrane protein essential for virus assembly
Source: Nat Commun. 2022 Aug 5;13:4399. doi: 10.1038/s41467-022-32019-3 (PMC9355944; doi:10.1038/s41467-022-32019-3)
Supplement: Supplementary file 3 — Description of Additional Supplementary Files [file 41467_2022_32019_MOESM3_ESM.pdf]

Description of Additional Supplementary Files

File Name: Supplementary Movie 1
Description: Conformational changes between the long and short forms of M protein dimer. The movie was generated using Morph Conformations in UCSF Chimera.
