# Supplementary material for: Structure of SARS-CoV-2 membrane protein essential for virus assembly
Source: Nat Commun. 2022 Aug 5;13:4399. doi: 10.1038/s41467-022-32019-3 (PMC9355944; doi:10.1038/s41467-022-32019-3)
Supplement: Supplementary file 5 — Reporting Summary [file 41467_2022_32019_MOESM5_ESM.pdf]

nature portfolio

Corresponding author(s): Umeharu Ohto, Toshiyuki Shimizu

Last updated by author(s): Jul 8, 2022

Reporting Summary

Nature Portfolio wishes to improve the reproducibility of the work that we publish. This form provides structure for consistency and transparency in reporting. For further information on Nature Portfolio policies, see our Editorial Policies and the Editorial Policy Checklist.

Statistics

For all statistical analyses, confirm that the following items are present in the figure legend, table legend, main text, or Methods section.

n/a	Confirmed	
☐	☒	The exact sample size (*n*) for each experimental group/condition, given as a discrete number and unit of measurement
☐	☒	A statement on whether measurements were taken from distinct samples or whether the same sample was measured repeatedly
☒	☐	The statistical test(s) used AND whether they are one- or two-sided<br>*Only common tests should be described solely by name; describe more complex techniques in the Methods section.*
☒	☐	A description of all covariates tested
☐	☒	A description of any assumptions or corrections, such as tests of normality and adjustment for multiple comparisons
☐	☒	A full description of the statistical parameters including central tendency (e.g. means) or other basic estimates (e.g. regression coefficient) AND variation (e.g. standard deviation) or associated estimates of uncertainty (e.g. confidence intervals)
☒	☐	For null hypothesis testing, the test statistic (e.g. *F*, *t*, *r*) with confidence intervals, effect sizes, degrees of freedom and *P* value noted<br>*Give P values as exact values whenever suitable.*
☒	☐	For Bayesian analysis, information on the choice of priors and Markov chain Monte Carlo settings
☒	☐	For hierarchical and complex designs, identification of the appropriate level for tests and full reporting of outcomes
☒	☐	Estimates of effect sizes (e.g. Cohen's *d*, Pearson's *r*), indicating how they were calculated

Our web collection on statistics for biologists contains articles on many of the points above.

Software and code

Policy information about availability of computer code

Data collection	Cryo-EM data were collected with the Serial EM 3.8 or EPU software.
Data analysis	MotionCor2 (implemented in RELION), CTFFIND4 (implemented in RELION), RELION (3.1), Chimera (1.13.1), ChimeraX (0.91), COOT (0.8.9.2), PHENIX (1.19.2-4158), Pymol (1.8.x), cryoSPARC (v3.0, v3.2), GROMACS (2019.6)

For manuscripts utilizing custom algorithms or software that are central to the research but not yet described in published literature, software must be made available to editors and reviewers. We strongly encourage code deposition in a community repository (e.g. GitHub). See the Nature Portfolio guidelines for submitting code & software for further information.

Data

Policy information about availability of data

All manuscripts must include a data availability statement. This statement should provide the following information, where applicable:

- Accession codes, unique identifiers, or web links for publicly available datasets
- A description of any restrictions on data availability
- For clinical datasets or third party data, please ensure that the statement adheres to our policy

All data needed to evaluate the conclusions in the paper are present in the paper and/or Supplementary Figures and Table. Additional data and resources related to this paper may be requested from the authors. The low-resolution cryo-EM map of M protein dimer in the absence of antibody has been deposited in the Electron Microscopy Data Bank (EMDB) under accession codes EMD-31976 [https://www.ebi.ac.uk/pdbe/entry/emdb/EMD-31976]. Cryo-EM maps and related structure

March 2021

coordinates of M/Fab-E and M/Fab-B complexes have been deposited in the EMDB and Protein Data Bank (PDB) under accession codes EMD-31977 [https://www.ebi.ac.uk/pdbe/entry/emdb/EMD-31977], PDB: 7VGR [http://doi.org/10.2210/pdb7VGR/pdb] and EMD-31978 [https://www.ebi.ac.uk/pdbe/entry/emdb/EMD-31978], PDB: 7VGS [http://doi.org/10.2210/pdb7VGS/pdb], respectively. For referenced structures, cryo-EM structure of SARS-CoV-2 ORF3a and structure of anti-Ghrelin receptor antibody under accession codes PDB: 6XDC [http://doi.org/10.2210/pdb6XDC/pdb] and PDB: 6KS2 [http://doi.org/10.2210/pdb6KS2/pdb], respectively. The source data underlying Figures 4b, 4c, 4e and Supplementary Figure 1, 13 are provided as a Source Data file.

Human research participants

Policy information about studies involving human research participants and Sex and Gender in Research.

Reporting on sex and gender	N/A
Population characteristics	N/A
Recruitment	N/A
Ethics oversight	N/A

Note that full information on the approval of the study protocol must also be provided in the manuscript.

Field-specific reporting

Please select the one below that is the best fit for your research. If you are not sure, read the appropriate sections before making your selection.

- [x] Life sciences
- [ ] Behavioural & social sciences
- [ ] Ecological, evolutionary & environmental sciences

For a reference copy of the document with all sections, see nature.com/documents/nr-reporting-summary-flat.pdf

Life sciences study design

All studies must disclose on these points even when the disclosure is negative.

Sample size	Cryo-EM sample size are determined by the availability of microscope time and the number and quality of particles to obtain the reported structures. The sample size of each dataset is indicated in Table and the image-processing procedures. For protein expression and purification, cell culture sizes were determine by the need to attain target yields. For other experiment, sample size were not predetermined.
Data exclusions	Cryo-EM images were excluded based on CTF max resolution parameters. Particles in 3D classification in RELION or cryoSPARC software with poor structural features were excluded. These procedures are general practice in the field of cryo-EM single particle analysis.
Replication	Recombinant protein purifications were performed several times. SEC, pull-down and LLPS assays were reproduced at least two times. All attempts at replication were successful.
Randomization	Randomization was performed when calculating Fourier-shell correlation of half maps. For other experiments, randomization was not applied. The statistical consideration is not relevant to these experiment because of the nature of biochemical and structural experiment performed in this work.
Blinding	Blinding was not relevant to this study. Blinding is not technically or practically feasible for the experiments in this study.

Reporting for specific materials, systems and methods

We require information from authors about some types of materials, experimental systems and methods used in many studies. Here, indicate whether each material, system or method listed is relevant to your study. If you are not sure if a list item applies to your research, read the appropriate section before selecting a response.

Materials & experimental systems

n/a	Involved in the study	
[ ]	[x]	Antibodies
[ ]	[x]	Eukaryotic cell lines
[x]	[ ]	Palaeontology and archaeology
[ ]	[x]	Animals and other organisms
[x]	[ ]	Clinical data
[x]	[ ]	Dual use research of concern

Methods

n/a	Involved in the study	
[x]	[ ]	ChIP-seq
[x]	[ ]	Flow cytometry
[x]	[ ]	MRI-based neuroimaging

Antibodies

Antibodies used	Mouse monoclonal anti-SARS-CoV-2 M protein Fab fragmants YN7705, YN7717, YN7730, YN7743, YN7756 and YN7761. Mouse monoclonal antibody against HA-tag [HA-probe (F-7); sc-7392, Santa Cruz Biotechnology, dilution 1:10,000] was used for immunoprecipitation and western blotting. A rabbit polyclonal antibody for SARS-CoV-2 nucleocapsid protein (GTX135357, GeneTex, dilution 1:10,000) and secondary antibodies, anti-rabbit IgG (HRP) (NA934, GE Healthcare, dilution 1:10,000) and anti-mouse Ig (HRP) (Mouse TrueBlot Ultra; 18-8817-30, Rockland Immunochemicals, dilution 1:10,000), were used for western blotting. Anti-DDDDK-tag mAb (MBL, Cat# M185-3L, Lot# 015, dilution 1:2,000), Rabbit Anti-Mouse IgG H&L (HRP) (Abcam, Cat# ab6728, Lot# GR3383345-1, dilution 1:2,000) and Anti-DDDDK-tag mAb-HRP-DirecT (MBL, Cat# M185-7, Lot# 009, dilution 1:2,000) were used for western blotting.
Validation	YN7705, YN7717, YN7730, YN7743, YN7756 and YN7761 were generated in this study. For antibodies obtained from commercial vendors, we relied on information provided in the manufacturers' corresponding data sheets.

Eukaryotic cell lines

Policy information about cell lines and Sex and Gender in Research

Cell line source(s)	ExpiSf9 and Expi293F cells were purchased from ThermoFisher Scientific (cat# A35243 and A14635). NS-1 myeloma was purchased from ATCC (cat# TIB-18). 293T cells were purchased from the American Type Culture Collection (ATCC, ATCC CRL-3216).
Authentication	The cell lines were authentified by the manufacturers and no further authentification was performed.
Mycoplasma contamination	Not tested.
Commonly misidentified lines (See ICLAC register)	No commonly misidentified cells were used in this study.

Animals and other research organisms

Policy information about studies involving animals; ARRIVE guidelines recommended for reporting animal research, and Sex and Gender in Research

Laboratory animals	Female Balb/c mice, 6 weeks of age, were maintained at temperature and humidity ranges of 22 to 26 degree Celsius and 40% to 60% humidity, respectively under a 12-h light, 12-h dark cycle.
Wild animals	No wild animals were used in the study.
Reporting on sex	Because of their less aggressive behaviors and hence the easy maintenance in the cage, female mice were used in the study.
Field-collected samples	No field collected samples were used in the study.
Ethics oversight	All animal experiments conformed to the guidelines of the Guide for the Care and Use of Laboratory Animals of Japan and were approved by the Kyoto University Animal Experimentation Committee.

Note that full information on the approval of the study protocol must also be provided in the manuscript.
